# Effective transcatheter valve implantation after pulmonary homograft failure: A new perspective on the Ross operation

**DOI:** 10.1016/j.jtcvs.2008.08.072

**Published:** 2009-07

**Authors:** Johannes Nordmeyer, Philipp Lurz, Victor T. Tsang, Louise Coats, Fiona Walker, Andrew M. Taylor, Sachin Khambadkone, Marc R. de Leval, Philipp Bonhoeffer

**Affiliations:** a UCL Institute of Child Health and Great Ormond Street Hospital for Children, London, United Kingdom; b The Heart Hospital, London, United Kingdom

**Keywords:** CPEX, cardiopulmonary exercise, MRI, magnetic resonance imaging, PA, pulmonary artery, PPVI, percutaneous pulmonary valve implantation, RV, right ventricle, RVOT, right ventricular outflow tract

## Abstract

**Objective:**

The Ross procedure offers good autograft function and low reoperation rates for the neoaortic valve; however, the rate of conduit dysfunction in the right ventricular outflow tract remains a concern. This study assessed percutaneous pulmonary valve implantation in this setting.

**Methods:**

We retrospectively analyzed outcomes of 12 patients (mean age 28 ± 5 years) referred for percutaneous pulmonary valve implantation to treat right ventricle–pulmonary artery conduit failure 11.1 ± 3.3 years after Ross procedure.

**Results:**

Percutaneous pulmonary valve implantation was feasible in all 12 patients, with no procedural complications (procedure time 99 ± 16 minutes, fluoroscopy time 21 ± 6 minutes). Right ventricular outflow tract gradient during catheterization and pulmonary regurgitant fraction on magnetic resonance imaging fell after valve implantation (gradient 34 ± 6 to 14 ± 3 mm Hg, *P* < .01, regurgitant fraction 20% ± 6% to 2% ± 1%, *P* < .05). After restoration of right ventricular outflow tract function, indexed right ventricular end-diastolic volume decreased (91 ± 13 to 78 ± 12 mL · beat^−1^ · m^−2^, *P* < .01) and maximal cardiopulmonary exercise performance improved (peak oxygen consumption 25.4 ± 2.3 to 30.8 ± 3.0 mL · kg^−1^ · min^−1^, *P* < .01). During follow-up (18.8 ± 4.6 months), there was 1 device explantation (restenosis). The probabilities of freedom from right ventricular outflow tract reoperation were 100% at 1 year and 90% at 3 years.

**Conclusions:**

Percutaneous pulmonary valve implantation provides an effective transcatheter treatment strategy to prolong the lifespan of right ventricle–pulmonary artery conduits after the Ross procedure, reducing the reoperation burden on patients with aortic valve disease.

The pulmonary autograft replacement of aortic valve (Ross procedure)^1^ represents an attractive surgical option for aortic valve disease, especially for younger patients. Because it uses the patient's own pulmonary valve as an aortic autograft, advantages include a favorable hemodynamic profile, the potential for adaptive growth,^2^ and the avoidance of anticoagulation therapy and its associated risks.^3^ In addition, the autograft function has been shown to be acceptable in several studies reporting midterm outcomes for this procedure in different patient populations.^3-7^ A major drawback of this procedure, however, is that the necessary placement of a conduit from the right ventricle (RV) to the pulmonary artery (PA) converts “single-valve disease” into “two-valve disease.” Although the orthotopic positioning of the RV-PA conduit may provide a reasonable longevity,^8^ the rate of RV-PA conduit dysfunction remains a significant concern, with an estimated freedom from reoperation or restenosis (peak RV outflow tract [RVOT] gradient exceeding 30 mm Hg) of only 64% at 6 years in a pediatric cohort.^6^ We report our experience with percutaneous pulmonary valve implantation (PPVI)^9,10^ as a transcatheter treatment option for RV-PA conduit dysfunction in this setting.

## Materials and Methods

### Patients

Of the 176 patients who underwent PPVI with the current device design (January 2003–November 2007), we retrospectively examined the outcomes of 13 patients who had RV-PA conduit dysfunction after the Ross procedure. One patient with repaired complex left ventricular outflow tract obstruction (coarctation with valvular and supravalvular aortic stenosis) was excluded from the analysis, because PPVI was performed only as part of a palliative strategy in the context of multiorgan failure. Most of the remaining patients (10/12) had the Ross procedure performed outside our institutions. Clinical indications for PPVI included RV hypertension with significant RVOT obstruction, significant pulmonary insufficiency, and RV dilatation or failure as described previously.^10^

PPVI was performed under general anesthesia at Great Ormond Street Hospital for Children, The Heart Hospital, and Harley Street Clinic (London, UK). The ethics committees at these institutions approved the study protocol, and written, informed consent was obtained from patients and parents as appropriate.

### Echocardiography

On echocardiography, peak RVOT gradient and RV systolic pressure (estimated from tricuspid regurgitant jet velocities) were obtained from continuous-wave Doppler traces (VIVID 7; GE, Medical Systems, Milwaukee, Wis). Color flow mapping of the RVOT and branch pulmonary arteries was used for qualitative grading of the degree of pulmonary regurgitation (0 absent, 1 trivial, 2 mild, 3 moderate, and 4 severe).

### Cardiopulmonary Exercise Testing

Ten of 12 patients underwent cardiopulmonary exercise (CPEX) testing before and early after PPVI (median 20 days after PPVI, range 2–381 days). The remaining 2 patients did not undergo CPEX testing because of pulmonary hypertension (n = 1) and nonmedical reasons (n = 1). A ramp protocol was performed on a mechanically braked bicycle ergometer (ergometrics ER 900 PC; ergoline GmbH, Bitz, Germany). Peak oxygen consumption was derived from respiratory gas analysis during CPEX testing.^12^

### Cardiac Magnetic Resonance Imaging

Cardiac magnetic resonance imaging (MRI) was performed for 10 of 12 patients before and early after PPVI (median 7 days after PPVI, range 1–364 days). The remaining 2 patients did not undergo cardiac MRI because they had pacemakers inserted. The MRI scans were performed at 1.5 T (Symphony Maestro Series and Avanto; Siemens Medical Systems, Erlangen, Germany). Retrospective gated steady-state free-precession cine images of the heart and arterial flow data with a flow-sensitive gradient echo sequence were acquired.^12^ Thereafter, ventricular volumes, pulmonary and aortic regurgitant fractions were calculated with the Argus analysis work package (Siemens Medical Systems).

### Follow-up Assessment

During structured follow-up, echocardiographic and chest radiographic investigations were performed immediately after PPVI and at 1 month, 3 months, 6 months, 1 year, and yearly thereafter. Echocardiography was used to assess the hemodynamic situation; chest radiography was used to screen for structural integrity of the stent.

### Statistical Analysis

Data are expressed as mean ± SEM unless otherwise specified. Two paired samples were analyzed with paired Student *t* tests or Wilcoxon matched pairs tests as appropriate. Multiple comparisons were performed with repeated measures analysis of variance and subsequent post hoc analysis (Bonferroni correction) as appropriate. Probability of freedom from reintervention was obtained by use of Kaplan–Meier plots. Statistical testing and data analysis were performed with SPSS version 11 (SPSS Inc, Chicago, Ill) and GraphPad InStat 3 Demo (Graphpad Software, Inc, La Jolla, Calif).

## Results

### Baseline Characteristics

Twelve patients (28 ± 5 years old, 21–98 kg) underwent PPVI for RV-PA conduit failure 11.1 ± 3.3 years after Ross procedure (Table 1). The indications for PPVI were predominant RV-PA conduit stenosis in 6 of 12 patients (50%), predominant conduit regurgitation in 4 of 12 (33%), and mixed conduit dysfunction in 2 of 12 (17%) (Table 1). The failed conduits comprised homografts in 11 of 12 cases (92%, first homograft n = 8, reconstructed first homograft n = 1, second homograft n = 2) and a porcine valve conduit in the remaining case (8%).

In the aortic position, 11 of 12 patients (92%) had the original autograft in place with no significant incompetence (median aortic regurgitant fraction 3%, range 0%–17%). One patient (8%) had a 19-mm St Jude mechanical valve (St Jude Medical, Inc, St Paul, Minn), which was inserted because of early autograft failure 1 year after the Ross procedure.

### Procedural Details

**Figure 1:**
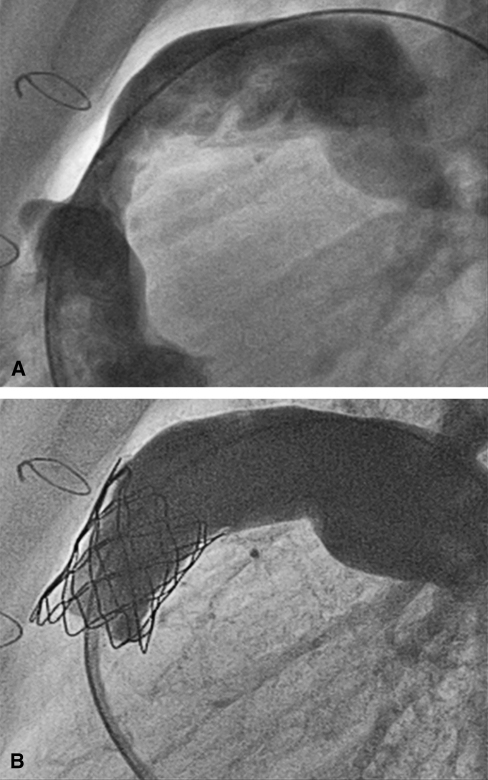
Angiographic appearance before (A) and immediately after (B) successful percutaneous pulmonary valve implantation.

PPVI was successful in 12 of 12 patients (100%; Figure 1), with no procedural complications (procedure time 99 ± 16 minutes; fluoroscopy time 21 ± 6 minutes). Prestenting with a bare-metal stent (Max LD; ev3 Endovascular, Inc, Peripheral Vascular, Plymouth, Minn) was performed in 2 of 12 patients (17%), particularly in the context of early second homograft failure, 1.5 years and 3.5 years after surgery.

### Hemodynamic Outcome

After PPVI, the invasively measured peak RVOT gradient, and RV-systemic pressure ratio fell acutely (RVOT gradient 34 ± 6 to 14 ± 3 mm Hg, *P* < .01, RV–SP ratio 0.68 ± 0.07 to 0.42 ± 0.03, *P* < .01). Likewise, pulmonary regurgitant fraction measured on cardiac MRI fell after PPVI (20% ± 6% to 2% ± 1%, *P* < .05).

On cardiac MRI, the indexed RV end-diastolic volume decreased (91 ± 13 to 78 ± 12 mL · beat^−1^ · m^−2^, *P* < .01; Table 2). Other parameters did not show statistically significant differences from before to after PPVI (Table 2).

### Functional Outcome

CPEX testing revealed improved indices of maximal cardiopulmonary exercise performance (peak oxygen consumption 25.4 ± 2.3 to 30.8 ± 3.0 mL · kg^−1^ · min^−1^, *P* < .01, maximum workload achieved 128 ± 11 to 150 ± 13 W, *P* < .01; Table 2). In addition, the anaerobic threshold increased after restoration of RVOT function (13.6 ± 1.3 to 15.0 ± 1.3 mL · kg^−1^ · min^−1^, *P* < .05; Table 2).

### Reintervention and Reoperation

**Figure 2:**
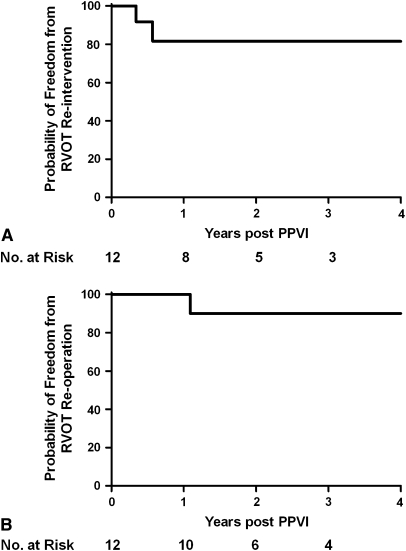
A, Freedom from right ventricular outflow tract *(RVOT)* reintervention was 81.5% at both 1 and 3 years. B, Freedoms from right ventricular outflow tract reoperation were 100% at 1 year and 90% at 3 years. *PPVI,* Percutaneous pulmonary valve implantation.

During a mean follow-up of 18.8 ± 4.6 months, 2 of 12 patients (17%) had further events. Both patients had a good initial result from PPVI but had RVOT obstruction recur because of stent fractures and were treated with second PPVI at 4 and 7 months, respectively. Subsequently, 1 of the 2 patients required third PPVI at 3 years, when further hemodynamically relevant stent fractures occurred. The other patient had restenosis in the context of medically treated endocarditis; however, the actual cause of restenosis could not be unequivocally identified. This patient underwent reoperation and device explantation at 13 months at another institution. In this series, the probabilites of freedom from RVOT reintervention were 81.5% at 1 and 3 years (Figure 2, *A*), respectively, and the freedoms from RVOT reoperation were 100% at 1 year and 90% at 3 years (Figure 2, *B*).

In all other patients (10 of 12, 83%), there was sustained hemodynamic improvement at latest follow-up relative to immediate postprocedural results. This improvement was demonstrated by echocardiography, which also showed preserved pulmonary valve competence (Table 3).

## Discussion

The search for the optimal surgical treatment for aortic valve lesions in young patients remains a significant challenge. Ideally, a replacement valve should offer native hemodynamic performance, the potential for adaptive growth,^2^ freedom from anticoagulation therapy^3^ (which has important implications for patient compliance), and good longevity. In this respect, the autograft valve comes close to the ideal, and it is superior to such other current options as bioprosthetic and mechanical valves. The Ross procedure continues to be questioned as a treatment option for patients with aortic valve disease, however, because of the potential for autograft dilatation or failure but in particular because of the need for RV-PA conduit. This study showed PPVI to provide a novel, effective transcatheter treatment strategy to prolong the lifespan of surgically placed RV-PA conduits in this scenario. Such a minimally invasive treatment for managing conduit dysfunction justifies a further review of the Ross procedure, particularly for young patients, who probably benefit most from the advantages of the autograft option.

In this series, we had good procedural success and did not see procedural complications with PPVI. This finding is related to the advantageous anatomic substrate in patients after the Ross procedure of circumferential RV-PA conduits placed into undistorted anatomy of the RVOT and the PAs. These anatomic features compare favorably with our experience with congenital right heart lesions^10^, in which the underlying RVOT anatomy often makes device implantation challenging, and in very rare cases even impossible. From a technical perspective, most patients with RV-PA conduit failure after the Ross procedure therefore qualify for PPVI. Particular care should be taken to prevent damage to the reimplanted coronary arteries, because their proximal course could be at risk from external compression during percutaneous valve deployment, although we have not seen this in our own experience. Careful preprocedural imaging assessment of the reimplanted coronary arteries in relation to the RVOT should therefore be performed. This imaging should involve noninvasive 3-dimensional imaging modalities (eg, cardiac magnetic resonance or computed tomography) in addition to standard angiography during catheterization.

Importantly, RVOT function was restored in all patients after PPVI, with significant reductions in pulmonary stenosis and regurgitation. A slight residual gradient across the RVOT may be found with this technique, however, because the device cannot always be expanded to its intended dimensions as a result of the varying geometry and distensibility of the failed RV-PA conduits (eg, the impact of asymmetric calcifications). The mean size of the original RV-PA conduits in this series revealed no evidence supporting patient–prosthesis mismatch; nonetheless, this possibility cannot be fully excluded. It is important to note that use of the current device is restricted to RV-PA conduits larger than 14 × 14 mm^11^ to achieve a sufficient valve orifice area. Therefore, patients who have received RV-PA conduits smaller than the suggested dimension (eg, those operated on at a very young age) thus do not qualify for PPVI. In our overall experience of PPVI, prestenting with bare-metal stents and postdilatation with high-pressure balloons has resulted in less residual gradients, leading to lower reintervention rates.^11^

The restoration of RVOT function was accompanied by changes in MRI and CPEX parameters, suggesting an objective improvement in cardiac function after relief of adverse RV loading conditions.^12^ These data represent the first description of early functional outcome after RVOT reintervention in the Ross population without the confounding effects of cardiopulmonary bypass. Long-term ventricular response and other outcome parameters, however, cannot be foreseen at present.

The only indication for reintervention in this series of PPVI was recurrent RVOT obstruction. Overall, 3 repeated PPVI were performed in 2 patients and was an effective treatment option for early device failure caused by stent fracture, the most common follow-up complication in our experience,^13^ resulting in further prolongation of the lifespan of surgically placed conduits.^14^ RVOT reoperation was performed in 1 patient, 13 months after PPVI. The surgery was performed at another institution for what was described as restenosis after medical treatment of endocarditis, although the actual cause of the gradient across the device could not be unequivocally identified at surgery.

### Limitations

Our study highlights an important treatment option for patients after the Ross procedure; however, it represents a retrospective analysis in a relatively small patient population. Most patients were referred to us from different institutions. All follow-up investigations were performed and read in an unblinded fashion. Although our study has demonstrated immediate functional benefits from this procedure, its long-term utility remains to be investigated.

## Conclusions

RV-PA conduit dysfunction after the Ross procedure can be successfully treated with PPVI. This may help decrease the cumulative surgical burden in the lifetime management of left ventricular outflow tract lesions and thus justifies a further review of the Ross operation, particularly for young patients.

## Figures and Tables

**Table 1:** Clinical characteristics of patients with percutaneous pulmonary valve implantation after Ross procedure

Case	Sex	Age (y)	Time since Ross (y)	RV outflow tract	RV-PA conduit lesion
1	M	42	Unknown	Homograft (2nd) 21 mm	Predominant stenosis
2	M	22	4	Homograft (2nd) 20 mm	Predominant stenosis
3	F	12	5	Homograft 18 mm	Mixed dysfunction
4	M	20	8	Homograft (reconstructed)	Mixed dysfunction
5	M	38	2.5	Homograft 23 mm	Predominant stenosis
6	F	26	5	Homograft 24 mm	Predominant stenosis
7	M	11	7	Homograft 19 mm	Predominant stenosis
8	M	13	10	Homograft 16 mm	Predominant regurgitation
9	M	71	34	Porcine valve conduit (unknown)	Predominant stenosis
10	F	24	8	Homograft (unknown)	Predominant regurgitation
11	F	15	8	Homograft 20 mm	Predominant regurgitation
12	M	48	31	Homograft (unknown)	Predominant regurgitation

*RV,* Right ventricle; *PA,* pulmonary artery.

**Table 2:** Functional outcome

Parameter	Before	After	*P* value
Cardiac magnetic resonance imaging			
Pulmonary regurgitant fraction (%)	20 ± 6	2 ± 1	<.05
RV end-diastolic volume (mL · beat^−1^ · m^−2^)	91 ± 13	78 ± 12	<.01
RV end-systolic volume (mL · beat^−1^ · m^−2^)	41 ± 10	34 ± 11	.06
Effective RV stroke volume (mL · beat^−1^ · m^−2^)	39 ± 3	43 ± 2	.11
RV ejection fraction (%)	59 ± 4	62 ± 5	.43
Cardiopulmonary exercise testing			
Peak oxygen consumption (mL · kg^−1^ · min^−1^)	25.4 ± 2.3	30.8 ± 3.0	<.01
Maximum workload (W)	128 ± 11	150 ± 13	<.01
Anaerobic threshold (mL · kg^−1^ · min^−1^)	13.6 ± 1.3	15.0 ± 1.3	<.05
Respiratory exchange ratio	1.10 ± 0.04	1.10 ± 0.02	.95

All data are mean ± SEM. *RV*, Right ventricle.

**Table 3:** Echocardiographic follow-up

	Peak RVOT gradient (mm Hg)	Pulmonary regurgitation
Preoperative	55.6 ± 7.8	2.2 ± 0.5
Postoperative	33.0 ± 3.0	0.4 ± 0.3
Latest follow-up	40.2 ± 5.9	0.5 ± 0.2
*P* value		
Preoperative vs postoperative	<.05	<.05
Postoperative vs follow-up	.50	.88

All data are mean ± SEM. *RVOT,* Right ventricular outflow tract.
